# Precise control of operating conditions in tribotesting with respect to trace humidity and contact temperature

**DOI:** 10.1016/j.mex.2021.101362

**Published:** 2021-04-24

**Authors:** Pontus Johansson, Kalle Kalliorinne, Pär Marklund, Marcus Björling

**Affiliations:** Division of Machine Elements, Luleå University of Technology, Luleå SE-97187, Sweden

**Keywords:** Humidity controller, Thermodynamic simulation, Tribology, Pin-on-disc

## Abstract

Research in tribology are often connected to tribosystems operating in specific environments, where climate chambers are needed for tribotesting to resemble the environmental conditions in the real application. Although the effect of humidity on the tribological performance of many materials and lubricants is evident, many studies are conducted without sufficient systems to accurately monitor and control the humidity level throughout testing. In this paper, a humidity controlling system was developed to enable continuous monitoring and precise control of the humidity at trace moisture levels. The climate controller was validated in a tri-pin-on-disc tribometer with excellent performance and can be fitted to most climate chambers. To further improve the control of operating conditions during tribotesting, a thermodynamic simulation of the contact temperature was developed.

• The developed climate controller is a simple and cost-effective method to accurately monitor and control the humidity in a climate chamber at trace moisture levels.
• The portable design of the humidity controller enables use with most climate chambers and enclosed tribometers.
• To have better control over the temperature in the sliding interface during testing, a thermodynamic simulation method was used to estimate contact temperature between sliding bodies from near-contact temperature measurements and the measured friction forces.

## Graphical abstract

**Figure:**
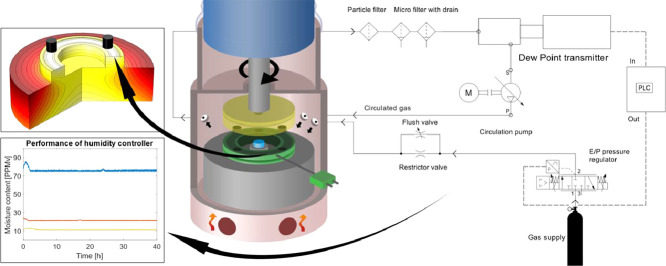

Specifications table

**Table:** 

Subject Area:	Engineering
More specific subject area:	Polymer composite tribology
Method name:	Climate control for tribotesting
Name and reference of original method:	Not applicable
Resource availability:	Software: PLC software, Logo! Soft Comfort - https://new.siemens.com/global/en/products/automation/systems/industrial/plc/logo/logo-software.html Simulation software, COMSOL Multiphysics - https://www.COMSOL.com/ Hardware: PLC, LOGO! 8 - https://new.siemens.com/global/en/products/automation/systems/industrial/plc/logo.html E/P pressure regulator, Sentronic LP 617 - https://www.emerson.com/en-us/catalog/asco-617 Dew point hygrometer, Easidew online - https://www.processsensing.com/en-us/products/easidew-online-dew-point-hygrometer.htm Eccentric gas pump, SPC 570 EC - https://schwarzer.com/pages_en/bauart.php?g=1&s=6 Kuhnke flow restrictor - https://productfinder.kuhnke.kendrion.com/en/flow-regulators-and-check-valves/

## Introduction

Many papers have proven the remarkable effect of sliding environment on friction and wear of various materials and lubricants, where the type of gas and humidity can greatly change the behaviour of friction and wear [1], [2], [3], [4]. In particular, the authors, among a few other researchers, have shown that the trace moisture content in the environmental gas has a significant effect on friction and wear of polymers and polymer composites [5], [6], [7], [8].

Tribometers are usually designed to suit a wide variety of configurations and users. Therefore, specific needs such as accurate humidity control in trace moisture, i.e. extremely dry, environments can be hard to find in a commercial tribometer. From literature, several different methods have been described in various levels of detail. Some of the described climate control systems are quite complex and likely very expensive [6,9], with accurate humidity measurements and possibilities to increase and decrease humidity. Other methods can simply be to supply gas at a given rate to keep humidity below a certain limit [10], purge a glovebox with a known gas for different durations to reach approximate levels of humidity [11] or insert a saturated salt solution in the climate chamber, where different salts provide different humidity levels [12]. The latter method is effective to maintain a certain humidity level, where the accuracy is known. However, only fixed humidity levels are possible and in the range of about 3 to 98% relative humidity. Most of the other described methods seem to need more or less manual adjustments or calibrations prior to testing to reach target humidity, where the accuracy of the humidity throughout testing is seldom specified.

Depending on the test setup, continuous monitoring and controlling of the humidity can be vital to guarantee correct humidity levels throughout testing. Absorbed or incorporated moisture in the sliding material can be released to the sliding environment due to frictional heat and wear and water vapor can migrate into the test chamber over time, increasing the humidity in the climate chamber. This slight rate of humidity increase can be devastating for precise humidity tests.

A climate controller was developed to monitor and control humidity at trace moisture levels with high accuracy, low gas consumption and inexpensive components. Key advantages of the controller are that it is closed-loop controlled, meaning no manual adjustments, and that it is built as a portable unit so that it can be used on most enclosed tribometers and climate chambers. The humidity controller was validated using an enclosed tri-pin-on-disc tribometer with high repeatability.

Contact temperatures in tribometers are by its nature extremely difficult to measure. Usually, temperatures measured close to the contact are used as an estimation of the interface temperature and to control the contact temperatures for elevated temperature conditions. However, the difference between measured near-contact temperature and actual contact temperature may vary depending on operating conditions and resulting coefficient of frictions. This could lead to misleading comparisons of tests due to variations in contact temperature. For better control of the contact temperature in the sliding interface a thermodynamic simulation model was developed.

## Description of the test rig

The tri-pin-on-disc was based on a Phoenix TE 92 rotary tribometer [13] where the TE 92/6 reservoir was used as a base for the test chamber. The operational limits of the TE 92, as specified in Ref. [13], can be seen in bold in Table 1, where derived limits for the tri-pin-on-disc setup are denoted with arrows.

**Table 1:** Operating limits of tribometer.

Parameter	Value
Rotational speeds	30–3000 rpm
• Linear sliding velocity	0.1–7.8 m/s
Load	20–10,000 N
• Contact pressure	0.2–66 MPa
Maximum friction torque	14 Nm up to 1500 rpm 7 Nm at 3000 rpm
• Maximum friction force	560 N up to 1500 rpm 280 N at 3000 rpm
Maximum temperature	200 °C (550 W heater power)

**Fig. 1:**
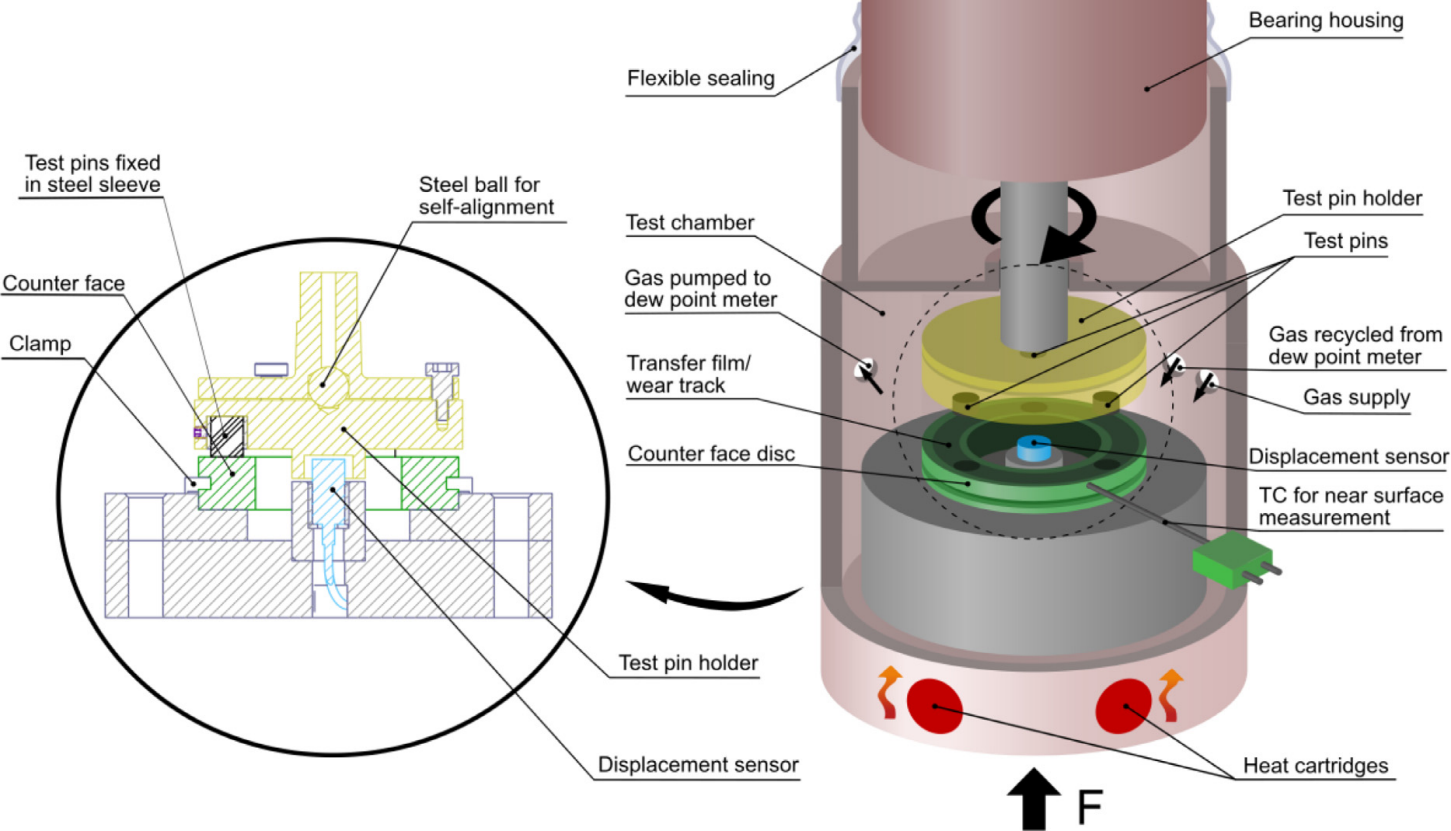
An illustrative description of the tri-pin-on-disc tribometer.

The developed tri-pin-on-disc configuration can be seen in Fig. 1 and mainly consists of a self-aligned test pin holder, an interchangeable counterface disc with a 25 mm mean sliding radius, a centered displacement sensor for wear measurements and a type K thermocouple (TC) for near-contact temperature measurements.

The test pin holder consists of two parts assembled with a centered steel ball between them, which allows the bottom part holding the three test pins to align against the counterface. The upper part has a machine taper to secure it to the rotating shaft. The torque is transmitted from the upper part of the test pin holder to the bottom by three symmetrically placed screws. Some amount of clearance between the upper part and screws exists to enable self-alignment. The test pins are 8 mm in diameter and have an initial length of 10 mm. The pins are put in slitted steel sleeves prior to mounting and then screwed tight to the pin holder by set screws. The test pin holder is designed to protect the displacement sensor from wear debris and also prevent the holder from damaging the sensor.

The tribometer test cell is enclosed by an o-ring sealed lid and a flexible sealing, consisting of a strip of plastic from a plastic bag and packing tape, between the lid and bearing housing to allow some vibrations and radial movement. Other types of sealing, such as a rotary shaft seal would cause interference with the friction measurements and limit the natural response of the sliding system. The rotating shaft is hollow on the TE 92 to enable the removal of the upper specimen. During tests, this hole is sealed on top using a threaded screw with a washer and o-ring. Three threaded holes were added to the reservoir in which tube connections were fitted. These were used for gas supply and gas circulation, which is explained in detail in the humidity controller section.

The displacement sensor is a high precision capacitance sensor, see specifications in Table 2. The sensor is of non-contact type, with no moving parts internally or externally. Therefore, the accuracy is not affected by mechanical hysteresis. Since the sensor is used to measure the micrometer-sized displacements due to wear rather than the absolute distance, the small non-linearity error can be neglected in this test setup. During operation, it was noted that the limiting performance parameter was the noise, where noise up to ±5 µm was observed at the full-scale output (FSO). This was lowered to a maximum noise of about ±1 µm by raising the sensor closer to the test pin holder with a shim. This increased the minimum signal strength and hence lowered the impact of noise from the surrounding.

**Table 2:** Specifications of displacement sensor.

Parameter	Value
Model	FE-925-CDT-02
Type	Capacitive displacement transducer (non-contact)
Range	0–2 mm
Non-linearity	< 0.1% at FSO, < 2 µm
Resolution	< 0.5 µm
Temperature	−25 to +200 °C

The thermocouple is inserted in a 1.5 mm in diameter hole in the side of the counterface disc. The hole is located 1.5 mm below the countersurface and reaches into the center of the sliding contact, providing close temperature measurements. The TE 92/6 reservoir is equipped with two heaters, illustrated in Fig. 1. These are closed-loop controlled to heat and maintain a specified near-contact temperature, as measured by the TC. No cooling unit is connected to the reservoir, instead, the reservoir together with the cylindrical base and the counterface works as a heat sink, dissipating excessive heat to the surrounding. Therefore, the near-contact temperature can only be controlled at elevated temperatures, where the minimum allowed temperature depends on the coefficient of friction and sliding velocity. For operating conditions used in the research paper [5], i.e. a sliding velocity of 2.2 m/s and a near-contact temperature of 80 °C, sufficient cooling was achieved for all test conditions where steady-state coefficient of friction of up to 0.18 was noted. For a higher coefficient of friction it was observed that the near-contact temperature exceeded 80 °C and an external cooling unit would have been needed to keep the near-contact temperature at the correct level.

## Approximation of contact temperature

**Fig. 2:**
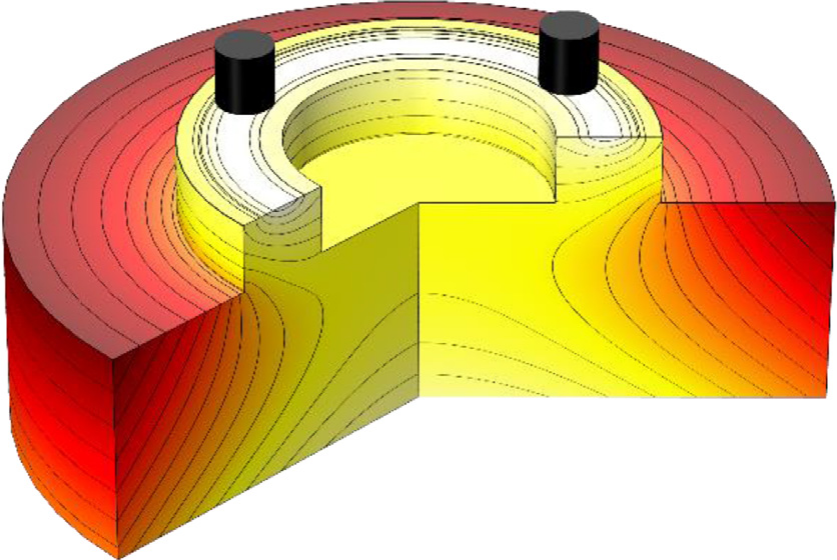
Simplified COMSOL model of the test setup.

A thermodynamic simulation with the setup depicted in Fig. 2 was carried out to approximate the steady-state contact temperature on the counterface disc. The setup is a simplified model of the lower part of the test setup seen in Fig. 1, consisting of the counterface disc and cylindrical base united to one solid. The modeling was done in the simulation software COMSOL Multiphysics inside the Heat Transfer Module, where all frictional work in the sliding interface was assumed to dissipate as heat.

**Fig. 3:**
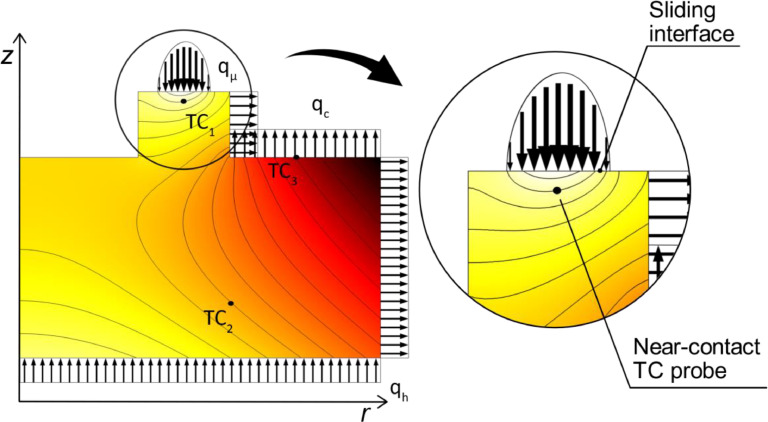
Thermodynamic simulation of axis symmetric model, where *z* denotes the symmetry axis and r the radius of the setup. Arrows indicate heat transfer in and out of the system and dots depicts positions of thermocouples.

The simulation was simplified as an axis symmetrical system, Fig. 3. Frictional heat is generated and conducted in the sliding contact, where the magnitude and parabolic distribution along the radius r of the contact surface of the corresponding heat flux $q_{\mu }$ is derived from test data and geometrical relations of the system,

(1) $$q_{\mu }\left(r\right)=\frac{\omega \;r\;\tau_{\mu }\;\cos^{-1}\left(\frac{{r_{p}}^{2}-{r_{d}}^{2}-r^{2}}{-2\;r\;r_{d}}\right)}{\pi \;A_{p}\;r_{d}}.$$

The input parameters in Eq. (1) are either known or directly measured in the test rig, where $\tau_{\mu }$ is the measured friction torque, ω is the angular velocity, $A_{p}$ the area of each test pin and $r_{d}$ the radius to the middle of the sliding surface on the counterface disc. The metal's thermal conductivity is generally significantly higher than polymer composites, wherein the relatively small part of the total heat flux going into the polymer composite pins was omitted from the simulation. Hence, all heat generated in the sliding interface was assumed to be thermally conducted by the disc in the model. Due to the parabolic heat flux distribution, Fig. 3, the contact temperature is varying across the transversal cross-section where the maximum temperature is found close to the radial center of the sliding contact. The sliding velocity increases towards the outer edge of the sliding contact, causing the heat distribution curve to be slightly skewed outwards from the rotational center.

The two heat cartridges depicted in Fig. 1 adds required heat to the system, giving rise to a heat flux $q_{h}$, which, for simplicity, is modeled as uniformly distributed to the bottom of the cylindrical base. The gas in the climate chamber is constantly moving because of pumping actions and the rotational movement of the test pin holder. The gas flow induces forced convection and potentially substantial heat losses. The convective heat flux $q_{c}$ is assumed to be uniformly distributed along the outer surfaces of the cylindrical base and counterface disc, where $q_{c}$ is a function of the convective heat transfer coefficient h and the temperature difference ΔT between the surfaces and ambient gas,

(2) $$q_{c}=h\Delta T.$$

The mass flow in and out of the small space formed between the inside of the counterface disc and the test pin holder during operation is considered small and hence the convective cooling of the inner surfaces is neglected and modeled as thermal insulators in the simulation model.

**Fig. 4:**
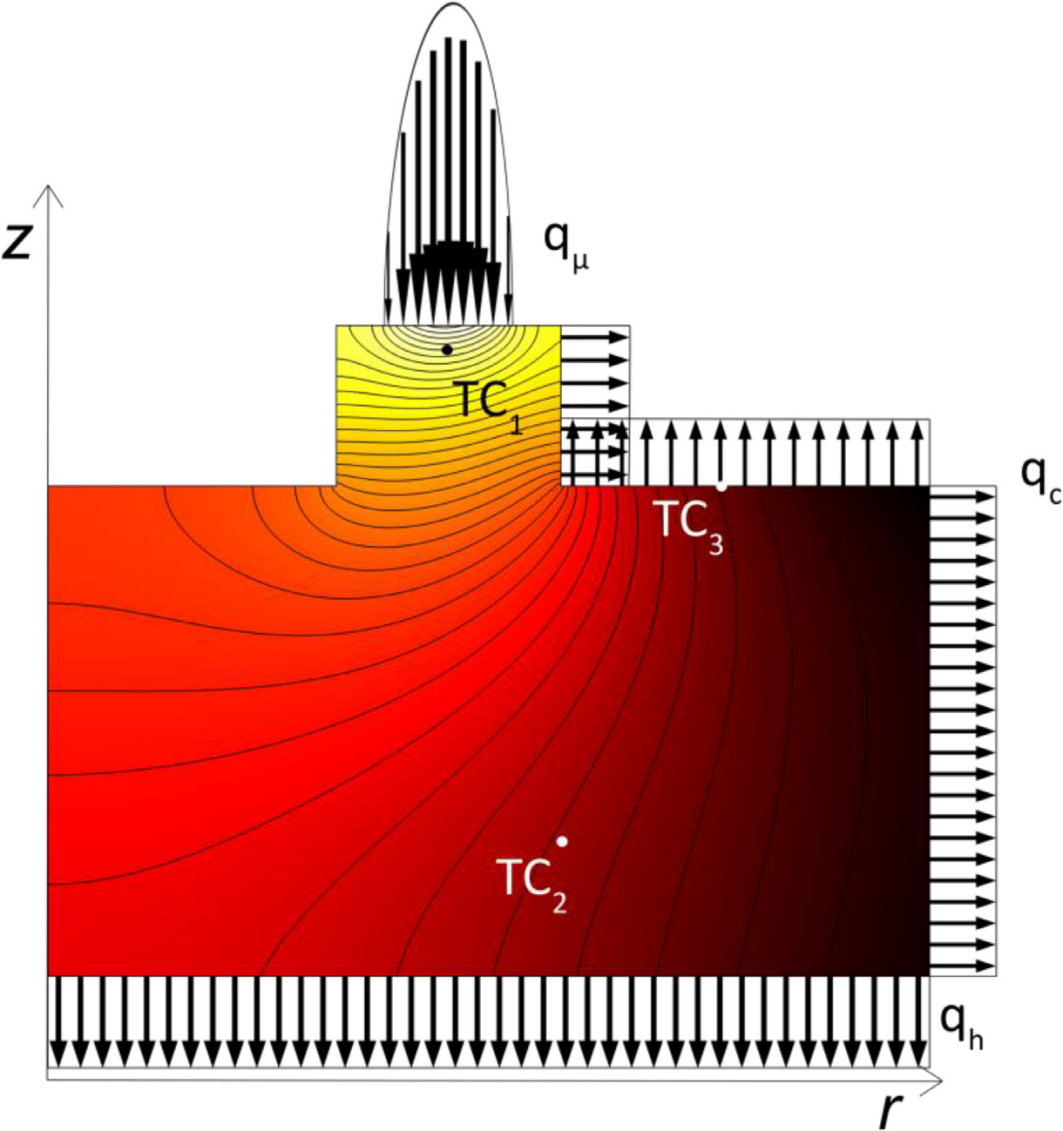
Thermodynamic simulation with higher friction torque used to validate the simulation model.

In accordance with the temperature control in the test rig, the main principle of the simulation model is to keep the temperature at the near-contact thermocouple $TC_{1}$, Fig. 3, to set value regardless of the coefficient of friction and corresponding heat flux $q_{\mu }$. The power from the heaters, and thus the heat flux $q_{h}$, are automatically adjusted for each simulation to fulfill this key criterion. Note that the allowed coefficient of friction is limited to 0.18 or less for the present temperature control system of the test rig due to the lack of a cooling unit as described in an earlier section. However, since $q_{h}$ is not limited to only positive values the model can simulate the use of a cooling unit and is therefore not limited to a coefficient of friction of 0.18 or less. The convective heat transfer coefficient hand the thermal conductivity K of the cylindrical base were unknown and without them a simulation conforming with the thermodynamics of the real test setup was impossible to find. Since the cylindrical base is fixed to the reservoir, K was assumed to be constant. Due to a fixed volume flow of 2 l/min in and out of the chamber the velocity of the gas inside the climate chamber should be relatively constant regardless of operating conditions and therefore, h was also assumed to be constant. Therefore, numerical optimization in the Optimization Module in COMSOL was performed to find suitable values for these parameters. The objectives in the optimization were two temperatures, one measured inside the cylindrical base by $\;TC_{3}\;$and the other measured the surface of the cylindrical base by $TC_{2}$, as depicted in Fig. 3. The temperatures were measured during tribotesting, where materials and operating conditions were similar to those described in the research paper [5]. The average friction torque for this test was calculated to 0.38 Nm which corresponds to a coefficient of friction of 0.05. The solver was limited to find a solution within a range of typical values of h for forced convection in air [14] and for typical values of K for steel [15]. Thermal radiation and other possible heat losses are indirectly included in the optimization as thermal convection. The values of h and K after optimization resulted in perfect conformity between simulated temperatures and the three measured temperatures. The simulation model was then validated using the resulting friction torque and temperature in $\;TC_{3}$ from a tribotest run at more severe conditions. The average friction torque for the validation test was 1.13 Nm, corresponding to a coefficient of friction of 0.15, and the temperature difference between $\;TC_{1}$ and $\;TC_{3}$ was measured to 17 °C compared to the test used for the optimization, where a difference of only 6 °C was measured. With the simulation solved for the higher friction torque, Fig. 4, the measured temperature in $\;TC_{3}$ was compared with the corresponding temperature in the simulation model, showing a difference of less than 1%.

As proven, the simulation model accurately estimates the contact temperature for tested operating conditions and coefficient of friction ranging between 0.05 and 0.15. Good estimations of the contact temperature are also expected for higher coefficients of friction, although the error will slightly increase with the coefficient of friction. This is mainly due to a simplification in the model that the gas temperature in the climate chamber is constant and independent of the coefficient of friction. However, in reality the gas temperature decreases with increased friction, which causes the error of $q_{c}$ to increase with coefficient of friction. Measurements of the gas close to the sliding contact during tests have shown that the gas temperature for tests with a coefficient of friction of 0.15 is about 10 °C lower than for tests with a coefficient of friction of 0.05. The insignificant error between measured and simulated temperature for the test at a coefficient of friction of 0.15 indicates that the deviation in gas temperature, and therefore also the increased error of $q_{c}$, does not affect the simulation results markedly.

**Fig. 5:**
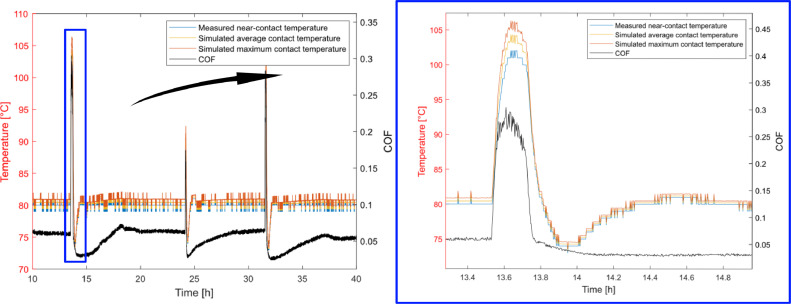
Measured near-contact temperature and simulated contact temperature for tribotest at steady-state conditions. Friction peaks are due to the specific wear behaviour of the tested polymer composite.

Temperature conditions and average friction torque from tests against the 34CrNiMo6 counterface disc in the research paper [5] were used as inputs for the simulation shown in Figs. 2 and 3. Friction torque and near-contact temperature data from one such test during steady-state conditions were used to simulate the average and maximum contact temperatures throughout a typical test where friction peaks occur. From the simulation, Fig. 5, it is evident that a low coefficient of friction results in insignificant differences between the measured near-contact temperature and simulated contact temperatures and that the difference linearly increases with the friction torque. From the simulation data the average contact temperature can be approximated by the linear equation

(3) $$T_{avg}=1.08\tau_{\mu }+T_{measured},$$

and the maximum contact temperature by

(4) $$T_{max}=1.97\tau_{\mu }+T_{measured},$$

where $T_{measured}$ is the near-contact temperature measured by thermocouple $TC_{1}.\;$As shown by Eqs. (3) and (4), the difference between the contact temperature and the measured temperature is negligible for these inputs, where the maximum contact temperature was less than 1 °C higher than the measured temperature at a friction torque of 0.38 Nm, equivalent to the steady-state coefficient of friction of 0.05 shown in Fig. 5, and about 5 °C higher at a friction torque of 2.5 Nm, equivalent to the peak coefficient of friction of 0.33 shown in Fig. 5. A similar trend applies for the simulated average contact temperature, only the difference is about half the size. The difference between simulated maximum contact temperature and measured temperature can also be seen in Figs. 3 and 4 for a coefficient of friction of 0.05 and 0.15 respectively, where the discrete change between each normalized temperature field curve is 0.5 °C.

Since the heat distribution is parabolic and the distance between the thermocouple and the sliding contact is much smaller than the radius of the test pins, it is reasonable that the temperature difference is small for the given inputs. Moreover, the increasing difference between the measured and simulated temperature with higher friction is also logical. Higher friction torque results in higher heat flux in the contact zone and less applied heat, if any, from the heaters, resulting in a steeper temperature gradient as can be seen by comparing Figs. 3 and 4.

It can be concluded that the difference between the maximum contact temperature and measured near-contact temperature under elevated temperature conditions can be neglected for mild sliding conditions. The temperature difference increase with the coefficient of friction but is only significant for very severe sliding conditions.

## Data acquisition

To minimize the post-processing time of the data from the displacement sensor and dew point transmitter, the existing data acquisition system (SLIM) of the TE92 tribometer was used. Two analog inputs with 12 bits resolution were available and could by small means be adjusted for the 0–10 V signals. The dew point meter was calibrated to the SLIM simply using a laboratory power supply and the displacement sensor was calibrated using a stand and precision gauge blocks with different heights.

## Humidity controller

**Fig. 6:**
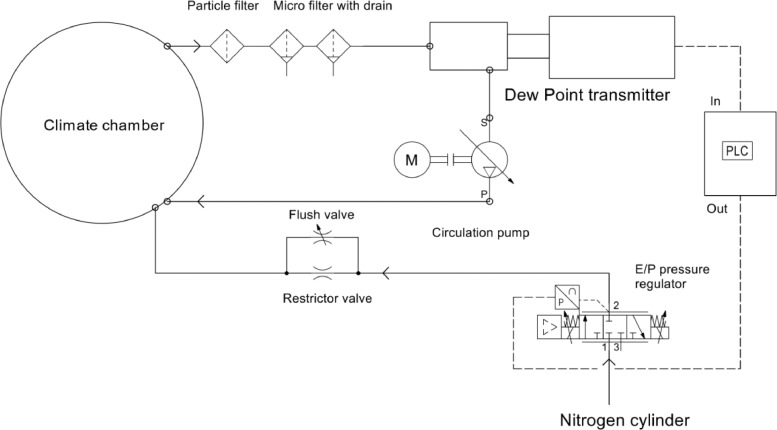
Schematics of the humidity controller.

The components used for the humidity controller can be seen in Fig. 6, where the key components are specified in Table 3.

**Table 3:** Specifications of components in the humidity controller.

Component	Product info
Dew point transmitter	Easidew online, −100 to +20 °C dew point range
Circulation pump	SPC 570 EC, 2 l/min
E/P pressure regulator	Sentronic LP 617, 0–6 bar
Filters	5, 0.3 and 0.01 µm
Restrictor valve	Kuhnke 0.1 mm orifice, approx. 0.34 Nl/min @ 7 bar
PLC	LOGO 8! with an analog output module
Tube	2-layer fluoropolymer tube, 6 × 4 mm

This system both monitor and actively control the humidity in the tribotest climate chamber. The humidity in the chamber is measured by circulating the environmental gas between the test chamber and a dew point meter by an eccentric diaphragm pump. The gas is pumped through a set of filters (5 to 0.01 µm) to protect the dew point meter from wear particles before entering the sampling block of the dew point meter. The dew point transmitter feeds a signal to both the data acquisition system of the test rig for continuous data logging and a Siemens LOGO8! PLC for feedback control of the humidity. Gas is supplied to the system through a restrictor valve to achieve sufficiently low gas flow. The pressure of the gas supplied to the restrictor valve is controlled by an electro-pneumatic (E/P) pressure regulator connected to a gas cylinder. A ball valve bypasses the restrictor valve to enable flushing of the test chamber to quicker reach target humidity after being exposed to the ambient environment.

For the specific tri-pin-on-disc test setup one restrictor valve with the 0.1 mm orifice gave slightly too high gas flow. This means that the pressure regulator only used a small portion of the total range during operation, leading to a somewhat rough controller. A lower flow allows the pressure regulator to operate in a wider pressure span which means that each discrete change in the controller output results in a smaller change in the flow and a smoother controlling system can be achieved. A restrictor valve with a smaller orifice would have been ideal, but in this case, a second restrictor valve of the same size connected in series was sufficient to reduce the flow due to the added pressure loss.

**Fig. 7:**
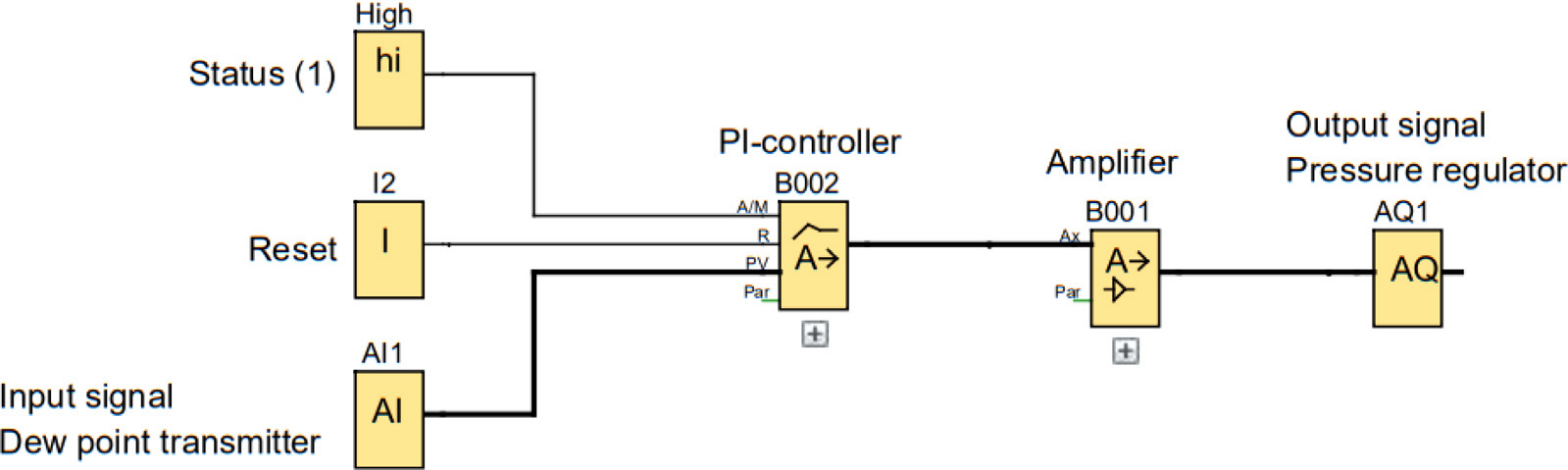
Schematic of PI-controller.

As illustrated in Fig. 6, the humidity controller is closed-loop controlled, using the signal from the dew point transmitter to regulate the pressure of the E/P pressure regulator, which in turn controls the flow of the supplied gas. The controller function is an in-built PI-controller in the software Logo8! Soft Comfort tuned to suit the system. The simple control function is illustrated in Fig. 7, where the function block (High) sets the proportional and integral (PI)-controller (B002) in automatic mode, (I2) can be connected to a physical button which resets the PI-controller, (AI1) is the analog signal from the dew point transmitter and (B001) is an amplifier used to offset the output signal (AQ1).

The setting of the controller parameters depends largely on the characteristics of the system, i.e., the nature of humidity, the amount of leakage of the test chamber and the maximum flow of supplied gas. The gas flow is determined from the pressure range of the pressure regulator and the size of the orifice in the restrictor valve. To be able to control the humidity by the change of supplied gas flow, some amount of leakage or a designated outlet port needs to exist in order to evacuate excessive moisture or draw in external moisture based on needs. This humidity controller was meant to be used continuously for long periods. Therefore, the gas consumption needed to be minimized, whereof the test setup was designed to allow a very limited leakage.

A controller system that could both handle the initial big difference between the process value (PV) and setpoint (SP), namely the controller error, during start-up and also smoothly tackle disturbances in the system proved to be hard to find due to the characteristics of the system. Reducing humidity takes time, reaching a moisture content of as low as 10 ppm takes several hours to reach from a humid state if a vacuum chamber and vacuum pump are not utilized. Water gets stuck in the filters and possibly in nooks in the test setup, making it a time-consuming process to reach trace moisture levels. To achieve a smooth and reliable controller at trace moisture levels the system needed to be flushed with nitrogen until the moisture content was reduced to about 70–90% of the target value. The initial flushing was executed in two steps; first flushing at full flow for some tens of seconds with the regulator set to about 0.05 to 1 bar just prior to fully sealing the climate chamber with flexible plastic and tape, and lastly by reducing the flow and keeping a slight overpressure after the chamber is fully sealed. The duration of the second step depended on the initial conditions and the setpoint and could vary between some tens of seconds to several minutes. The gas flow in the second step was adjusted with the ball valve by slowly opening it until the flexible plastic seal expanded slightly due to the overpressure in the climate chamber.

The Ziegler-Nichols method [16] was initially used to tune the humidity controller. The tuning with this method is basically done by setting the integration time to the maximum value to remove the integral gain and then increasing the proportional gain until oscillation occurs, namely the critical gain. The critical gain and resulting period of the oscillation are then used to calculate the proportional gain and integral time. Due to the high inertia of the humidity controlling system, the period of oscillation was up to several hours making the critical gain hard and time-consuming to find. Therefore, the Ziegler Nichols method was only used as a rough pre-tune to get initial parameter values. The controller was then fine-tuned by trial and error, which required ample understanding of the characteristics of the complete humidity controller and the impact of each of the controller parameters.

**Fig. 8:**
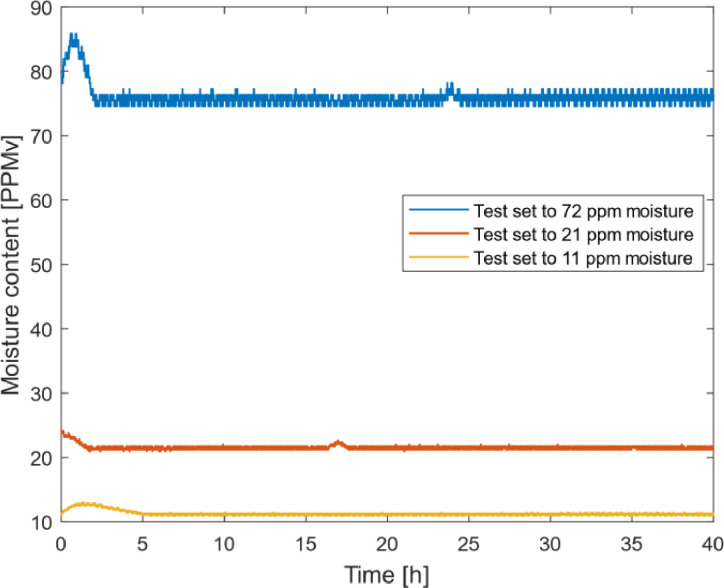
Performance of humidity controller with $K_{p}=10$ and $T_{i}=40$ min.

Due to the characteristics of the system, a short integral time $T_{i}$ enabled the controller to increase the output too much before the set point was reached, causing excessive over-shoot. A high proportional gain $K_{p}$ led to a fast controller but problems with oscillations and instability. After a lot of testing and tuning two satisfactory settings were found for different purposes. With $K_{p}=10$ and $T_{i}=40\;$ min, a relatively fast controller was achieved. With this setting, humidity changes were corrected for quickly and almost straight humidity curves could be achieved for the three tested humidity levels seen in Fig. 8 and a maximum error of less than 10%. The humidity was converted to moisture content in parts-per-million by volume (ppm_V_) using the saturation vapor pressure formula given by Huang [17].

**Fig. 9:**
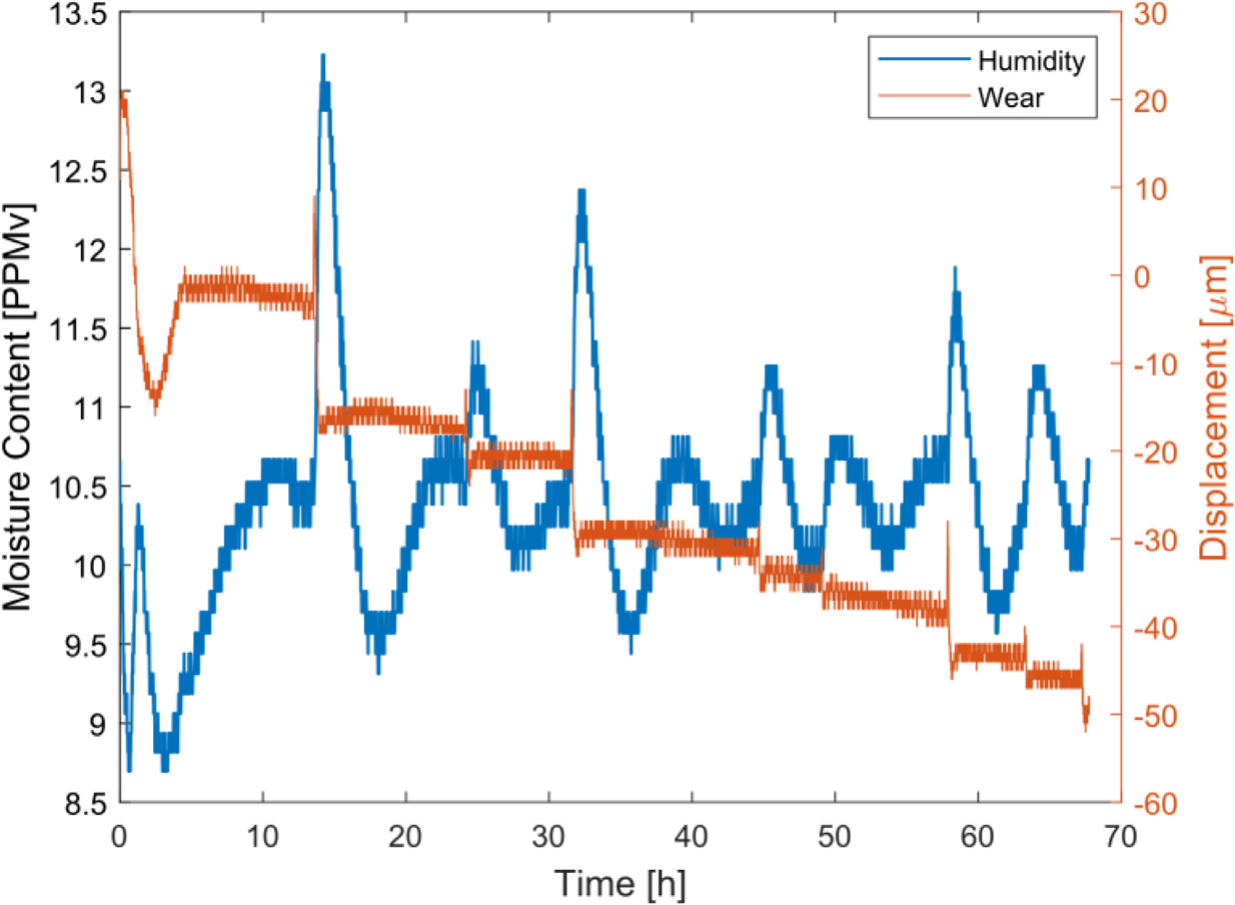
Obtained humidity changes due to tribological actions for controller settings $K_{p}=3$ and $T_{i}=10\;\min$.

A lower proportional gain of $K_{p}=3$ and shorter integral time $T_{i}=10$ min resulted in a slower controller with a more visible system response. The humidity could still be held within a few ppm_V_ of target humidity, but for this setting, signs of humidity influencing phenomena became evident. In Fig. 9, the increase of humidity could be directly connected to momentary periods of severe wear of a certain polymer composite as indicated by the sharp drops in the displacement curve. This could indicate that water molecules were incorporated or absorbed in the tested polymer composite.

The fast controller settings resulting in the straight humidity curves in Fig. 8 may seem like the best option for accurate control of the humidity. However, the more aggressive changes in the output together with the relatively long delay before changes are sensed by the dew point meter could cause unnoticed fluctuance in the humidity in the climate chamber. The output changes with the slow controller are smoother, which reduces possible fluctuance and by that creating a more reliable system.

An important modification to the PI regulator was to add a gain, B002 in Fig. 7, to the output signal to positively offset the signal to prevent the E/P pressure regulator to fully close. The dew point measurement is not instantaneous and it takes some time for humidity changes in the climate chamber to be registered by the dew point meter. A fully closed valve leads to zero over-pressure in the test chamber and humid air is sucked into the sliding environment causing unstable friction behaviour for materials sensitive to humidity. The gain was adjusted to keep the pressure regulator at a minimum opening at the minimum output, which eliminated this unwanted behaviour.

The humidity controller has been tested in a range of 10 to 100 ppm_v_ with excellent results. Humidity levels down to 5 ppm_v_ are most likely achievable but for levels above 100 ppm_v_ a humidifier is needed to get stable humidity. A simple and inexpensive solution can be used to humidify the climate chamber and still enable control by the flow of supplied gas. By putting a saturated salt solution in a wide-bottomed glass container with a gas-tight lid with two tube connectors, the gas can be humidified on its way back from the dew point meter by connecting the salt solution container between the circulation pump and climate chamber. However, this method would need some trial and error to choose an appropriate salt and sufficiently large surface area between the saturated salt solution and gas. One approach would be to choose a saturated salt solution [12] that provides a humidity higher, but as close as possible, to the target humidity and then stepwise increasing the exchange surface area until the rate of humidification is large enough but still controllable.

**Fig. 10:**
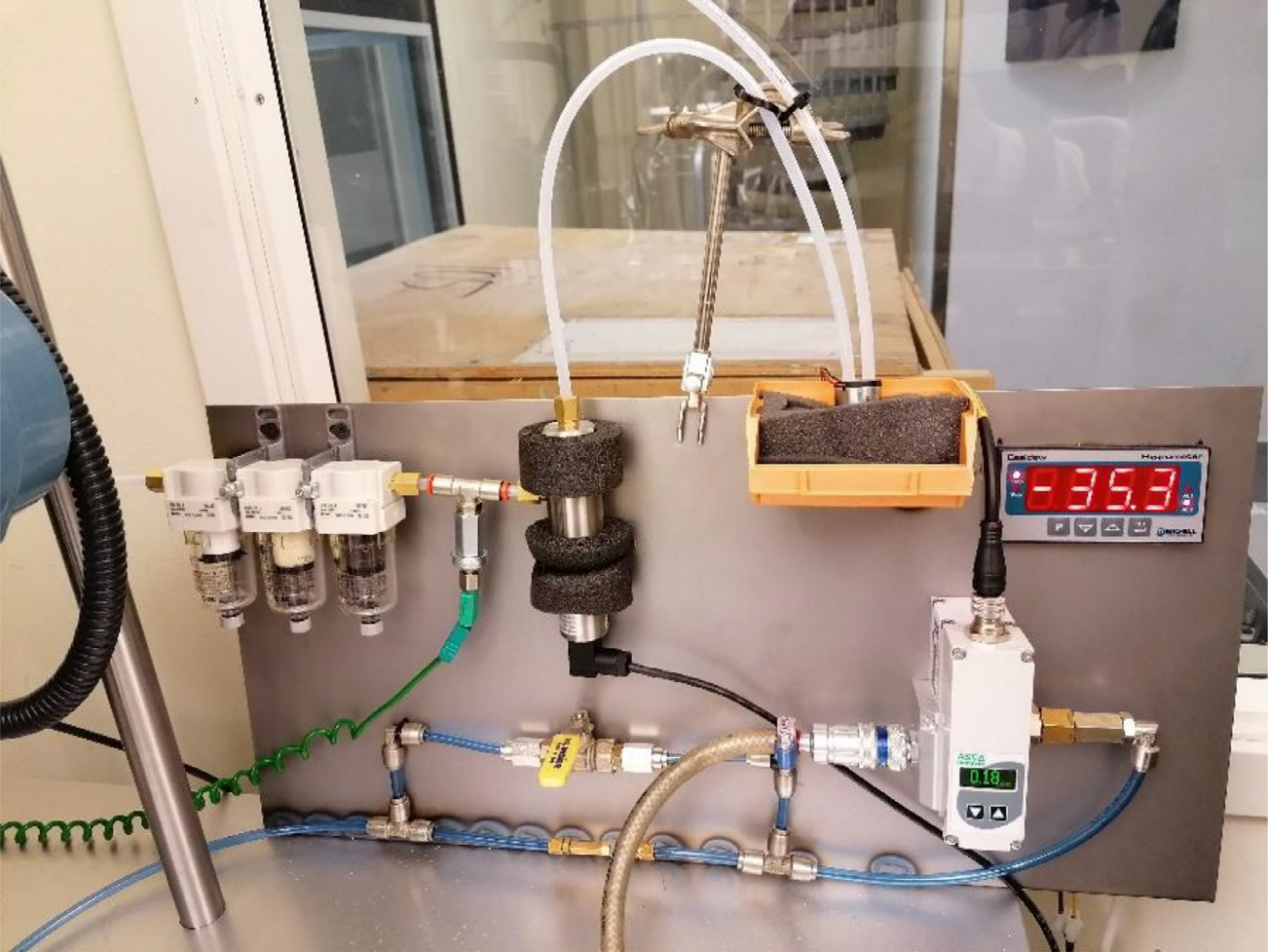
Final assembly of the portable humidity controller.

As seen in Fig. 10, the humidity controller is designed as a portable unit with all the equipment except the PLC mounted on a sheet of metal with table clamps. Therefore, the controller can easily be moved and used with other enclosed test rigs or climate chambers with minimal modifications. The humidity controller has only been tested with nitrogen but other inflammable gases can be used too. The low gas consumption makes it affordable to use more expensive gases such as helium.

The humidity controller could easily be upgraded to also measure the amount of oxygen in the test chamber by installing a trace oxygen sensor in series with the dew point meter. E.g., the thermocouple fitted between the filter rack and dew point meter, Fig. 9, has proven to be redundant in most cases and could therefore be replaced by an oxygen sensor.

## Concluding remarks

The developed method for controlling humidity at trace moisture levels has proven to be accurate, stable and well-functioning. Due to the simplicity and portability, the humidity controller can be moved and optimized to measure and control the humidity in most enclosed tribometers and climate chambers. Components can also be added or replaced to fulfill special requirements such as measuring the amount of oxygen in the gas, supply a mixture of two gases or control higher humidity levels than described in this paper.

For tribotesting, it is important to conduct tests under operating conditions similar to the actual application. Contact temperatures in sliding systems are often difficult to measure, wherefore thermodynamic simulations can be used to approximate the contact temperature from known parameters. The thermodynamic simulation method described in this paper has provided valuable information of the contact temperature and thermal transfer in and out of the test setup, e.g., the insignificant temperature difference between the near-contact thermocouple and the contact. The thermodynamic simulation model was validated with different inputs and temperature measurements from two separate locations to certify that the model is compliant with the real test setup. The results showed that the difference between measured near-contact temperature and contact temperature follows a linear relation with the friction torque, where Eqs. (3) and (4) can be used to approximate the average and maximum contact temperature using the friction torque and measured temperature as inputs. For test conditions promoting high friction coefficients, Eq. (3) can be used to calculate the difference between average contact temperature and measured near-contact temperature and use the result to compensate the temperature setting of the near-contact temperature to achieve the desired contact temperature.

Similar methodology can be used to develop thermodynamic simulation models for contact temperatures in other types of tribometers, given that near-contact temperatures are possible to measure and additional measurement points that can be used to optimize and validate the model.

## Direct submission or co-submission

Co-submissions are papers that have been submitted alongside an original research paper accepted for publication by another Elsevier journal

## Declaration of Competing Interest

The authors declare that they have no known competing financial interests or personal relationships that could have appeared to influence the work reported in this paper.
